# Cytokine dysregulation persists in childhood post Neonatal Encephalopathy

**DOI:** 10.1186/s12883-020-01656-w

**Published:** 2020-03-30

**Authors:** Zunera Zareen, Tammy Strickland, Victoria Mc Eneaney, Lynne A. Kelly, Denise McDonald, Deirdre Sweetman, Eleanor J. Molloy

**Affiliations:** 1 Discipline of Paediatrics, Trinity College, The University of Dublin, Dublin, Ireland; 2 Trinity Translational Medicine Institute (TTMI), Trinity College Dublin, Dublin, Ireland; 3 Paediatrics, Children’s Hospital Ireland (CHI) at Tallaght University Hospital, Dublin, 24 Ireland; 4 Paediatrics, National Maternity Hospital, Holles Street, Dublin, Ireland; 5 Paediatrics, Coombe Women and Infants University Hospital, Dublin, Ireland; 6 Neonatology, CHI at Crumlin, Dublin, Ireland

**Keywords:** Cytokines, Neonatal encephalopathy, Inflammation, Neurodevelopmental, Hypoxic-ischaemic encephalopathy

## Abstract

**Background:**

Cytokines are possible mediators of neuroinflammation and associated with adverse outcome in neonatal encephalopathy (NE). Our aim was to explore cytokine response in children with Neonatal Encephalopathy (NE) at school age compared to age-matched controls.

**Method:**

Follow up at school age, children who had NE and age-matched controls were assessed for their cytokine responses and neurodevelopment outcome. Pro- and anti-inflammatory cytokines in the serum, [Interleukin (IL)-1α, IL-1β, IL-2, IL-6, IL-8, IL-18, Tumor necrosis factor (TNF)-α, TNF β, Interferon (IFN)-γ, granulocyte-macrophage colony-stimulating factor (GM-CSF), vascular endothelial growth factor (VEGF), erythropoietin (EPO), IL-10 & IL-1RA] were measured at baseline and in response to in vitro stimulation with lipopolysaccharide (LPS: endotoxin).

**Results:**

GM-CSF, TNF-β, IL-2 IL-6 and IL-8 were significantly elevated at school age following NE (n = 40) compared to controls (n = 37). A rise in GM-CSF, IL-8, TNF-α, IL-1β, & IL-6 were seen in NE group following LPS stimulation. Relative LPS hypo-responsiveness was also noted in children with severe NE with IL-10, VEGF, EPO and TNF-β. Elevated TNF-β was associated with low gross motor scores on assessment at school age.

**Conclusion:**

School-age children post-NE had significantly altered cytokine responses to endotoxin compared to controls. TNF-β was associated with adverse developmental outcomes. This suggests the inflammatory process may persist into childhood and a longer therapeutic window may be available for neuroprotection therapies.

## Background

Neonatal brain injury such as Neonatal encephalopathy (NE) is an important cause of neonatal death and disability such as cerebral palsy. Inflammation combined with Hypoxia-ischemia (HI) play an important pathophysiological role in NE. The injury processes can persist for months and years and a tertiary mechanism of damage has been proposed, which includes inflammation and epigenetic changes [1], decreased plasticity and reduced number of neurons. Infants with NE have a persistent inflammatory response over the first week of life correlating with the degree of brain injury [2, 3]. Studies have shown that intermittent and sustained Inflammation demonstrated in preterm newborns may contribute to adverse neurodevelopmental outcome [4].

Pro-inflammatory cytokine expression within the brain, especially of Interleukin (IL)-1β and Tumor Necrosis factor (TNF)-α have been demonstrated following perinatal brain damage by pathogen triggers and hypoxic injury both in experimental models and the human neonates [5]. Neuroinflammation and IL-6, enhances the growth of neural precursors after neonatal brain injury [6]. Pro-inflammatory cytokines activate cytotoxic T cells and natural killer cells, which enhance cellular and tissue damage. This leads to cell proliferation, differentiation and cell death causing white matter changes and long-term neurological damage [7, 8].

Alternations in cytokines in NE during the neonatal period have been well described [9]. Cytokines including IL-6 and IL-16 in cord blood were associated with severity of neonatal encephalopathy defined by continuous electroencephalography [10]***.*** Elevated Interleukin (IL-6), noted in the first few hours after birth, in infants with NE who received TH was associated with death and abnormal neurodevelopmental outcome at 12 months of age [11]. In addition, levels of IL-6, IL-8, and vascular endothelial growth factor (VEGF) were also noted to be greater at 6–24 h in infants with moderate to severe NE compared to mild NE and were associated with abnormal neurological outcomes [1, 3, 12].

Therapeutic hypothermia remains the standard treatment available for moderate to severe neonatal encephalopathy. The concept of tertiary mechanism of damage has led to several research studies looking at alternative modalities of treatment that may not only reduces brain damage but also promote cell repair in the developing brain long after the insult. Certain immunomodulatory agents including EPO, melatonin and stem cell therapy have therapeutic potential and hold promise for neuroprotection [13].

We have previously demonstrated altered VEGF and Epo in the same cohort of babies with NE in the first week of life [2] and therefore followed them in childhood to assess immune function. We hypothesised that the inflammatory response persists in childhood and aimed to examine the cytokine profile of school aged children with Neonatal Encephalopathy in comparison to healthy age-matched controls. Understanding this persistent inflammatory mechanism could lead to safe and effective therapies to treat a developmentally disrupted brain long after the initial insult.

## Methods

### Ethical approval

Ethical committee approval was received from the Ethics Committee of the National Maternity Hospital, Dublin and the National Children Hospital, Tallaght, Dublin. Written informed consent was obtained from all parents of children enrolled in the study.

### Patient Groups

This study is a follow up of a previous cohort of infants with Neonatal encephalopathy recruited in the neonatal period and performed follow up at school age. The recruitment of these babies as neonates has been previously described [2]. The following groups were enrolled:

1. *Control group*: serum samples from healthy children age 4–7 years (n = 40) who attended the day ward and were undergoing phlebotomy as part of a day case procedure. These children were born following a full-term normal vaginal delivery with normal Apgar scores, had a normal neurological examination and postnatal course and had no underlying co-morbidities.
2. *NE group****:*** School age children at age of 4–6 years (n = 37) who were exposed to perinatal asphyxia (PA) according to the criteria of Huang et al [14], were included. Children with NE were classified using the modified Sarnat & Sarnat [15] as follows: number of infants with perinatal asphyxia but with no neurological signs (designated as grade 0 for study purposes, n = 2); mild NE (grade I, n = 11); moderate NE (grade II, n = 22); and severe NE (grade III, n = 2).

Fifteen of 24 children in the NE II/ III group had received therapeutic hypothermia (TH) in the neonatal period in accordance with the TOBY (Total Body Hypothermia for Neonatal Encephalopathy) criteria [16] and were treated for 72 h duration. Infants with congenital abnormalities, confirmed sepsis or evidence of maternal substance abuse were not enrolled [2].

### Development Questionnaire

The Ages and Stages Questionnaire was used as a screening tool to assess different domains of development including gross motor (GM), fine motor (FM), problem solving (PS), communication and personal social skills in children in the control group and children with NE. The age-appropriate development Ages and Stages Questionnaire (ASQ-3) was completed by the parents of school age children in both the NE group and the control group enrolled in the study. The age of the children was in the range of 4–6 years. All domains of development including gross motor, fine motor, problem solving, communication and personal social skills were examined. The parents completed the questionnaire at the clinic visit and any parental concerns were addressed. It took around 10–15 min for the parent to complete the questionnaire. A detailed history was taken, and neurological examination was carried out at the same visit.

### Blood sampling

Peripheral venous samples (1 mL) were collected into vacutainers containing sodium citrate. The sample was split into microtubes and treated with Vehicle (1 μl PBS) or Lipopolysaccharide (LPS) (10 ng/ml) at 37 °C for 1 h. Following incubation, the samples were centrifuged (1500 rpm at 4 °C × 10 min) and the supernatant removed, aliquoted and stored at − 80 °C for further analysis [2].

### Multiplex cytokine analysis

Interleukin-1 alpha (IL-1α, interleukin-1 beta (IL-1β), interleukin-2 (IL-2), interleukin-6 (IL-6), interleukin-8 (IL-8), interleukin-10 (IL-10), interleukin-18 (IL-18), interleukin-I receptor antagonist (IL-Ira), tumour necrosis factor-alpha (TNF-α), tumour necrosis factor -beta (TNF-β), interferon gamma (IFN-ϒ), vascular endothelial growth factor (VEGF), erythropoietin (Epo) and granulocyte macrophage/colony-stimulating factor (GMCSF) were analysed using a multi-spot 96-well, 14 spot custom human serum plasma plate which was customised for our study and selected cytokines by Meso Scale Discovery. The plate was then analysed on the SECTOR Imager and validated (Meso Scale Discovery, Rockville, MD, USA; www.mesoscale.com) [2].

### Statistical Analysis

Statistical analysis was carried out using the PASW statistical package version 24 (www.ibm.com/SPSS_Statistics). Continuous normally distributed data was displayed as means and standard deviations (SDs) and comparisons were made using the independent student-t test. Significance was achieved for values *p* = < 0.05. We used ANOVA-one-way analysis of variance for analysis of the cytokine results. Two-way analysis of variance was carried out to compare baseline and stimulated sample results. Spearman correlation was used to assess the association between the cytokine’s levels in children with NE and neurodevelopment outcome using ASQ-3.

## Results

### Clinical Characteristics

The study enrolled 77 school age children (age range 4–7 years), including controls (n = 40) and children following NE (n = 37). The Sarnat and Sarnat grades [15] were as follows: number of infants with perinatal asphyxia but with no neurological signs (designated as grade 0 for study purposes, n = 2); mild NE (grade I, n = 11); moderate NE (grade II, n = 22); and severe NE (grade III, n = 2). Fifteen children received therapeutic hypothermia (TH) in accordance with the TOBY (Total Body Hypothermia for Neonatal Encephalopathy) criteria [16] and they were treated for 72 h duration. Seventeen infants developed clinical seizures in the neonatal period and 13 had an abnormal MRI brain. There were no significant differences between the NE groups 0/I and II/III regarding gestational age, gender or birth weight (Table 1).

**Table 1:** Demographics of infants with NE

Variables	NE 0/1 (n = 13)	NE- II/III (n = 24)	*p*- value (95% CI)
**GA (wks)**^**a**^	39.7 (1.43)	40.5 (1.21)	0.06 (−1.4-0.03)
**BW (kgs)**^**a**^	3.48 (0.7)	3.61 (0.61)	0.31 (−4.7–0.13)
**Gender, male, n (%)**^**b**^	9 (76%)	14 (58%)	0.70
**Mode of delivery n (%)**^**b**^			
**LSCS**	7 (53)	11 (45)	0.70
**SVD**	3 (23)	7 (29)	0.73
**Inst**	3 (23)	6 (25)	0.73
**Apgar @1 min**^**c**^	5 (3–6)	2 (1–5)	0.003
**Apgar@5 mins**^**c**^	7 (5–8)	4 (2–7)	0.01
**Apgar@10 mins**^**c**^	7.5 (6–9)	5 (3–7)	0.01
**TH, n (%)**^**b**^	0 (0)	15 (62)	< 0.001
**Seizures, n (%)**^**b**^	0 (0)	17 (70)	< 0.001
**MRI- Abnormal, n (%)**^**b**^	0/0	13 (50)	< 0.001
**ASQ -GM**	56 (4)	48 (9)	0.03

*GA* gestational age, *BW* birth weight, *SVD* spontaneous vaginal delivery, *inst*. instrumental delivery, *TH* therapeutic hypothermia, *ASQ* ages and stages questionnaire, *GM* gross motor skill score. ^a^ For normally distributed data, mean ± SD is expressed, and the independent Student-t test was used for comparison, where *p* < 0.05 is significant with 95% confidence intervals (CI). ^b^ For binary variables, the χ2 test was used for comparison. ^c^ For skewed data, medians and IQRs are expressed and the Mann-Whitney U test was used for comparison

### Neurodevelopmental Outcome

Twenty-four children with moderate to severe NE (NE II/III) had the following outcomes on clinical review at school age as designated by their clinical teams: Cerebral Palsy with Gross Motor Function Classification System (GMFCS), level III-V (n = 5); epilepsy (n = 1); speech and language difficulty (n = 5); hearing deficit (n = 2); functional motor and co-ordination problems (n = 3).

The children in the NE group scored poorly in all domains compared to children in the control group, especially in gross & fine motor skills, communication and problem-solving (*p* value < 0.04). The scores for children with mild NE (NE 0/I) though in the normal range, were significantly lower when compared to children in the control group especially for fine motor and problem-solving skills. (NE 0/I v Control; FM: 58 vs 48, *p* value = 0.03; PS: 58 vs 55, *p* value =0.04, respectively). Similarly, on comparing the NE groups by severity, the NE II/III group scores were significantly low compared to NE 0/I group (NE II/III v NE 0/I: FM: 39 vs 48, *p* = 0.025; PS: 48 vs 55, *p* value = 0.036).

### Cytokines and Neurodevelopmental Outcome

Cytokine IL-6 was noted be significantly high on LPS stimulation in children with NE II/III who received TH and had abnormal neurodevelopmental outcome. Tumor Necrosis factor (TNF-β) is another pro-inflammatory cytokine which acts on different cells. In our study high levels of TNF-β in response to LPS stimulation were noted in school age children with NE. We also demonstrated association of high levels of cytokine TNF -β with low gross motor scores on developmental assessment at 5–6 years of age using the ASQ questionnaire (Rho 0.39, *p* value 0.02).

### Cytokines in children with NE versus Controls and Hypo responsiveness to LPS

**Fig. 1:**
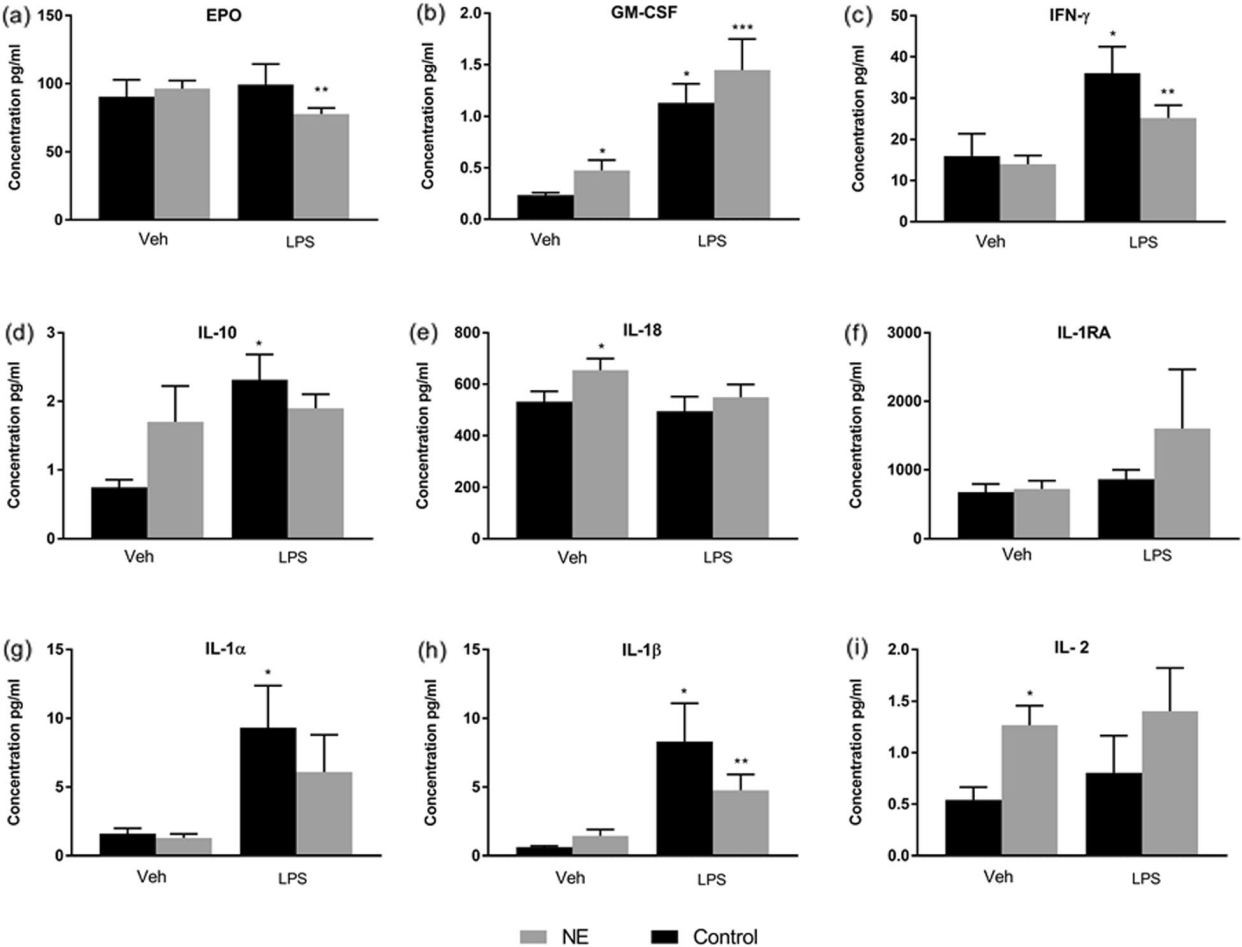
Cytokine response in school age children with NE compared to control at baseline and on LPS stimulation: Epo, GM-CSF, IFN-ϒ, IL-10, IL-18, IL-1ra, IL-1α, IL-1β and IL-2: Mean serum cytokine concentration expressed in pg./ml, GM-CSF = Granulocyte macrophage colony stimulating factor, IL = Interleukin, IL-1RA = Interleukin1-Receptor antagonist, IL-1β = Interleukin 1beta, IFN Ý = interferon gamma, IL = Interleukin, IL-1β = Interleukin 1beta, Vehicle or baseline, LPS = Lipopolysaccharide. * p value < 0.05 versus Veh/ Control; ** versus Veh/ NE

**Fig. 2:**
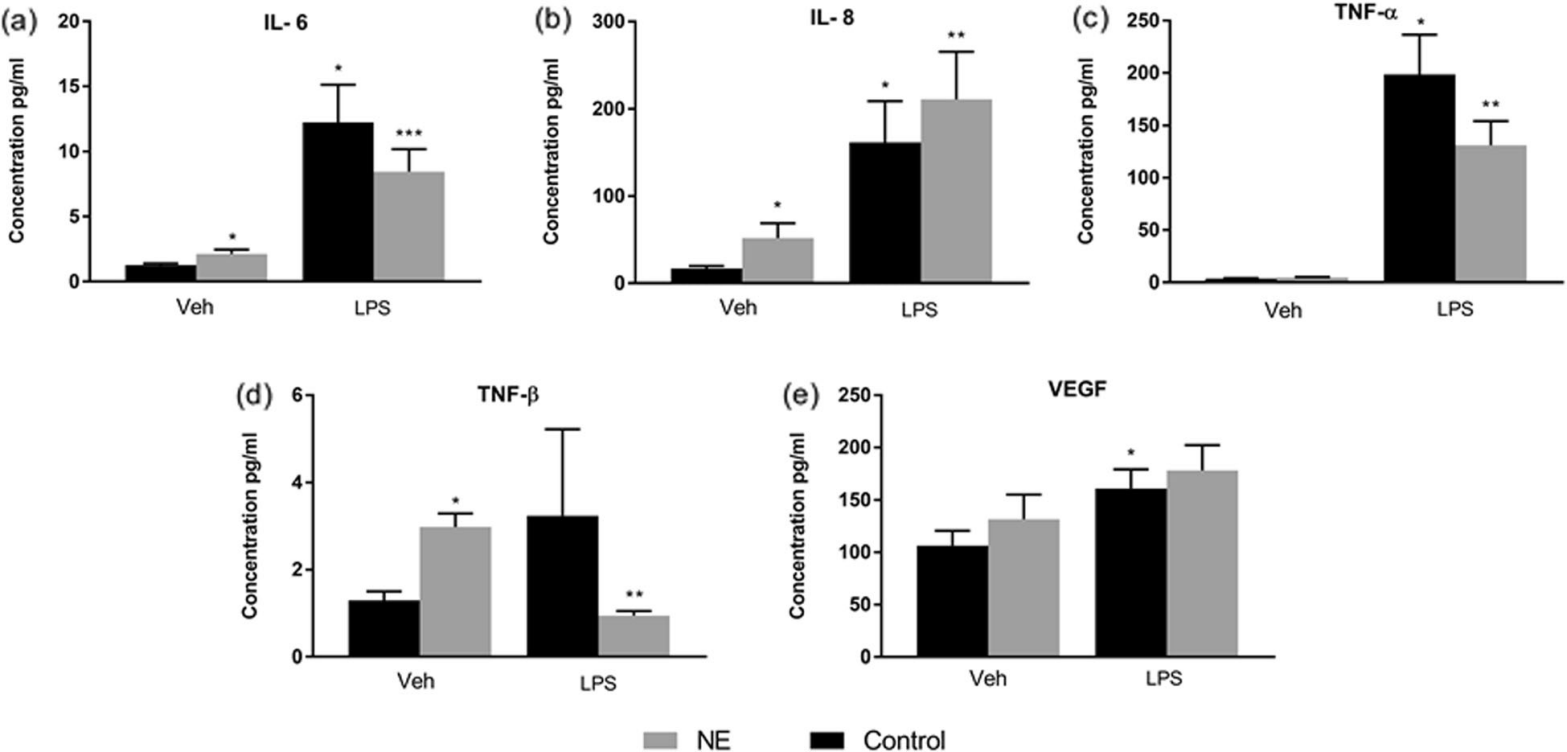
Cytokine response in school age children with NE compared to control at baseline and on LPS stimulation: Interleukin IL-6. Il-8, TNF-α, TNF-β and VEGF: Mean serum cytokine concentration expressed in pg/ml, TNF-α = Tumor necrosis factor alpha, VEGF = vascular endothelial growth factor. Veh = Vehicle or baseline, LPS = Lipopolysaccharide. * versus Veh Control p value < 0.05, ** versus veh/NE; *** versus all other parameters

GM-CSF, TNF-β, IL-2 IL-6 and IL-8, were significantly higher at school age (*p* value < 0.05) in children with NE compared to children in the control group (Table 2). In addition, on LPS stimulation a significant rise of cytokines GM-CSF, and IL-8 were seen in children with NE compared to controls (*p* value < 0.05). IL-6, IL-1 β and TNF-α were significantly increased in both groups of children with NE and controls (*p* value < 0.01) on LPS stimulation (Figs. 1 and 2).

**Table 2:** Cytokines levels in control group, NE0/I and NE II/III

Cytokines	Control (n = 40) (Mean)	NE (0/I) (Mean)	NE (II/III) (Mean)	*p* value (a)	*P* value (b)
**IL2**	0.54	1.18	1.35	**0.02**	**0.40**
**IL6**	1.27	2.03	2.31	**0.05**	0.73
**IL8**	16.77	37.99	77.67	0.11	0.39
**TNF α**	3.63	4.74	4.69	0.17	0.95
**GMCSF**	0.24	2.01	0.41	**0.02**	0.34
**IFN Y**	15.98	15.33	11.44	0.92	0.29
**EPO**	90.45	98.07	93.22	0.62	0.63
**IL10**	0.75	1.68	1.74	0.21	0.95
**IL 18**	532.57	628.01	683.14	0.16	0.56
**IL -IRA**	681.91	767.10	649.44	0.68	0.61
**IL-1α**	1.60	0.85	2.01	0.12	0.11
**IL-1β**	0.62	1.52	1.32	0.20	0.81
**TNF β**	1.29	3.17	2.63	**0.01**	0.42
**VEGF**	106.65	122.57	148.57	0.50	0.68

Means and p value using Student t-test. *IL* interleukin, *TNF* tumor necrosis factor, *IFN* interferon, *VEGF* vascular endothelial growth factor, *EPO* erythropoietin, *GMCSF* granulocyte colony stimulating factor. p (a)-p value Control vs NE 0/I and p (b) p value NE0/I vs NEII/III

**Fig. 3:**
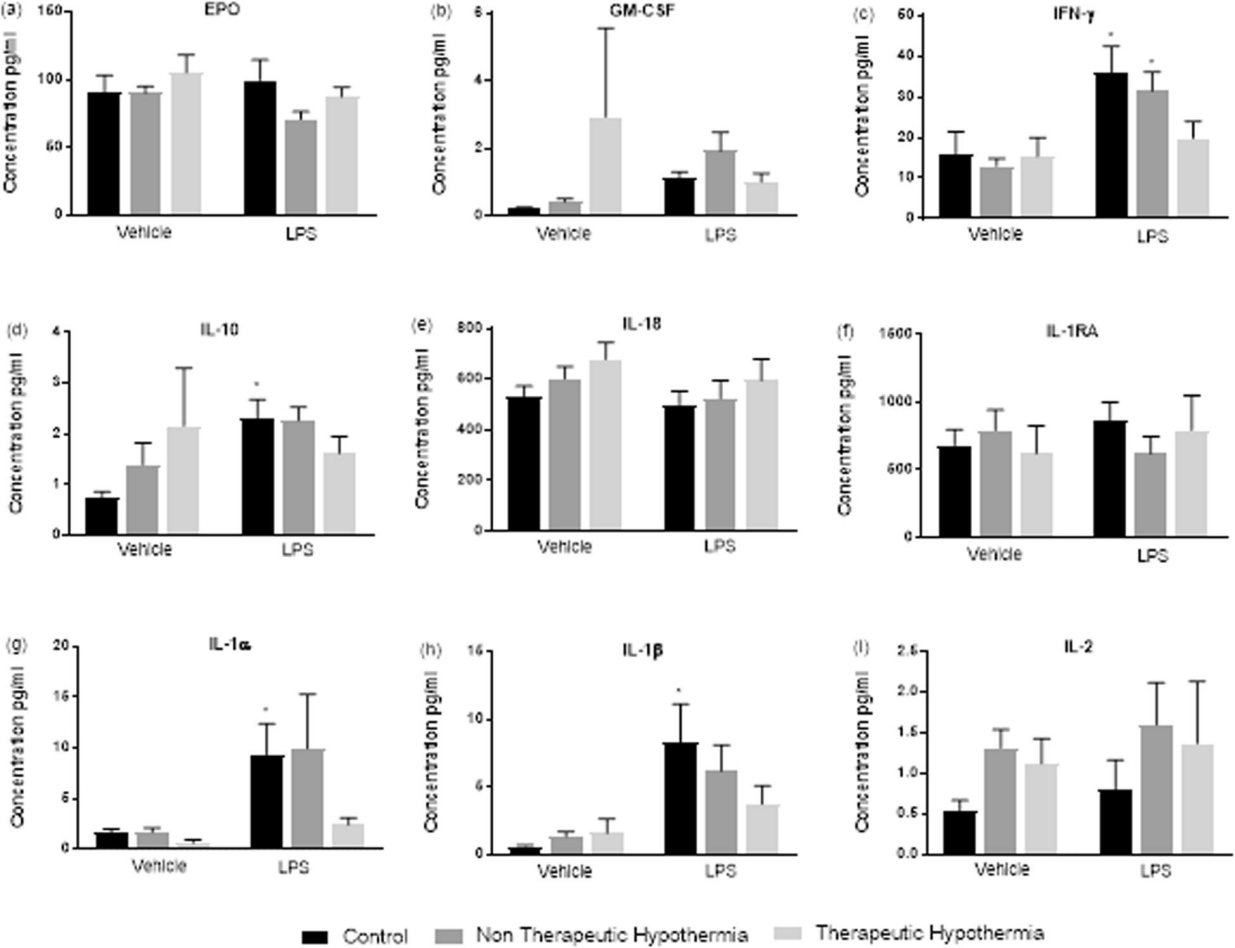
Cytokine response in children with NE who underwent Therapeutic Hypothermia (TH) vs NE without TH at baseline and on LPS stimulation: Cytokines EPO, GMCSF, IFN-ϒ, IL-2, IL-10, IL-18, IL-1ra, IL-1α, IL-1β: Cytokine concentration expressed in pg/ml Epo = Erythropoietin, GM-CSF = Granulocyte Macrophage Colony stimulating factor, IL = Interleukin, IFNÝ = Interferon gamma, IL-1RA = Interleukin 1 Receptor antagonist, Veh = Vehicle or baseline, LPS = Lipopolysaccharide. * *p* < 0.05 versus Veh Control

There was no increase in the pro inflammatory cytokines, IL-1α, IL-1β, IFN-ϒ and TNF-α, at baseline in the control group but these rose significantly when stimulated with LPS in vitro. There was no statistically significant increase in IL-10, IL-1Ra and VEGF in children with NE even on stimulation with LPS. LPS hypo-responsiveness was noted in Epo and TNF-β in children with NE. At baseline levels of both EPO and TNF-β rose slightly but on stimulation with LPS the levels were significantly reduced in children with NE compared to controls (Figs. 2 and 3).

**Fig. 4:**
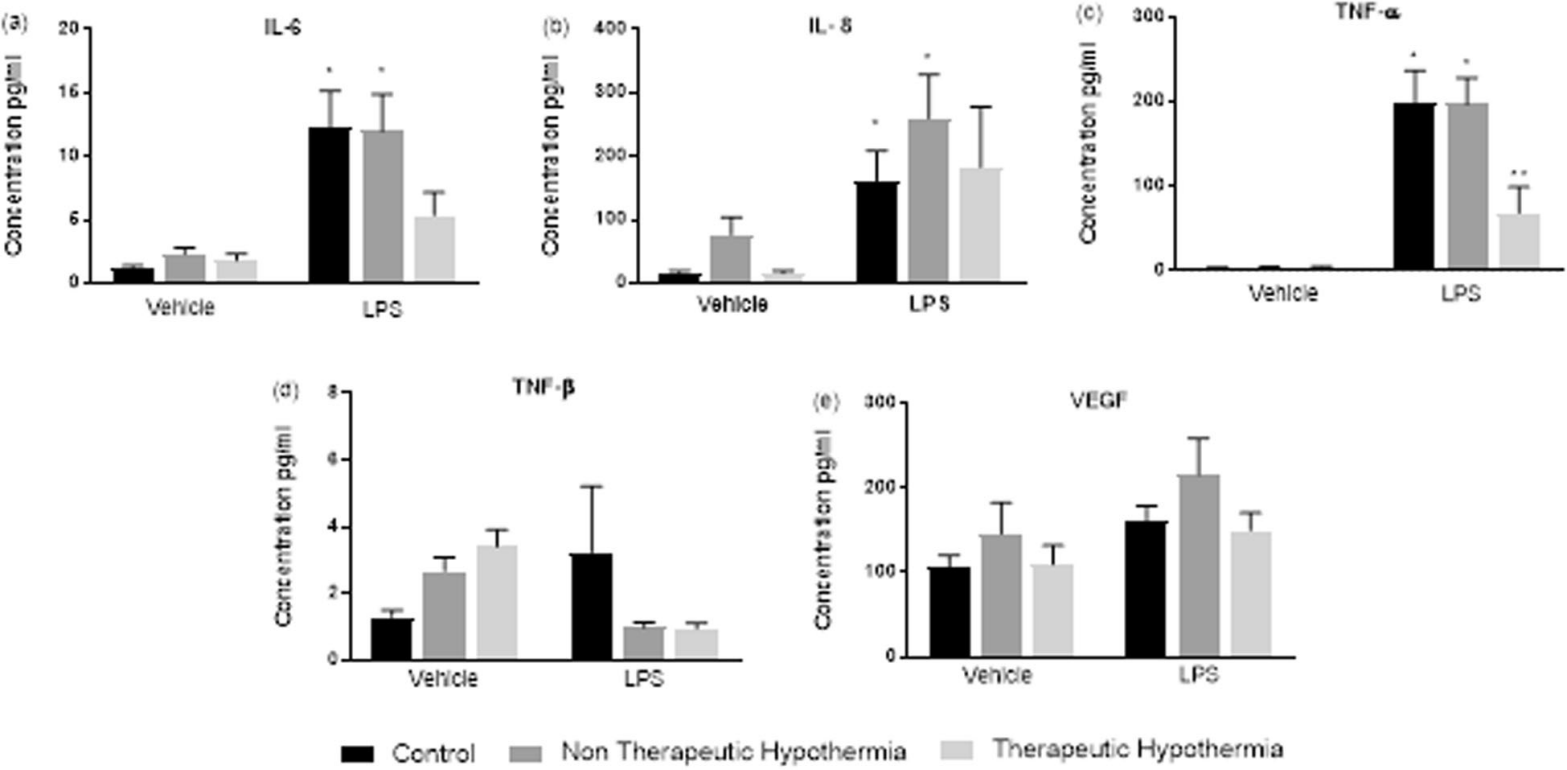
Cytokine response in children with NE who underwent TH vs NE without TH at baseline and on LPS stimulation: Interleukin IL-6. Il-8, TNF-α, TNF-β and VEGF: Cytokine concentration expressed in pg/ml, in children with NE and Therapeutic Hypothermia versus NE without TH. * *p* < 0.05 versus Veh Controls

Children who received TH had high levels of GM-CSF and IL-10 compared the control group (*p* < 0.05). However, on stimulation with LPS the levels of GM-CSF and IL-10 were noted to be reduced in the group of children who received TH compared to those without TH (Fig. 3). Similarly, decreased response to LPS in cytokines IL-6, IL-8, IL-1α, Il-1β, TNF-α and VEGF was demonstrated in children in the TH group compared to children who did not receive TH (Figs. 3 and 4).

## Discussion

We found a persistently abnormal cytokine response in children at school age following NE. GM-CSF, TNF-β, IL-2 IL-6, and IL-8, were significantly higher in children with NE compared to controls at baseline. GM-CSF and IL-8 rose significantly in children with NE in response to LPS stimulation compared to age-matched controls. Children at school-age following NE also had relative LPS hypo responsiveness in several cytokines demonstrating an altered immune phenotype compared with age-matched controls.

IL-8 was significantly elevated in school children with NE when compared with controls, and after in vitro treatment with an endotoxin (LPS) levels increased further. IL-8 was noted to be high in infants with NE when compared to normal infants and higher in those with severe encephalopathy and abnormal neuroimaging, compared to those with mild NE [3]. IL-8 measured before 96 h of age, to be a potent biomarker predicting death and abnormal neurological outcome in survivors of NE [9]. Elevated IL-8 levels in the group of children with NE, in our study compared to normal children demonstrates a persistently altered immune response at school age.

GM-CSF was also significantly elevated in children with NE compared to the controls. Interestingly increased response was also noted in the neonatal period in the same cohort of children with NE. GM-CSF activates neutrophils and macrophages and stimulates the production of other pro-inflammatory cytokines. GM-CSF production is stimulated by lipopolysaccharide (LPS), tumor necrosis factor-α (TNF-α), interleukin-1β (IL-1β), and IL-23. GM-CSF has been reported to high in infants with NE who were eligible for TH and in non-survivors at day 1 of life compared to survivors [3]. We also noted a similar response at school age in children with NE who underwent TH in the neonatal period. However, GM-CSF crosses the blood brain barrier and may be neuroprotective and reduced brain damage has been reported in previous experimental models of stroke [17].

IL-6 was noted be significantly high on LPS stimulation in children with NE II/III who received TH and had abnormal neurodevelopmental outcome. A study demonstrated that high levels of serum Il-6 at 24–48 h in a group of infants who received TH, were associated with abnormal MRI brain [18]. A meta-analysis identified serum IL-6 and serum IL-1β measured before 96 h of age as potential biomarkers in predicting abnormal outcome in infants with NE [9]. Elevated IL-6 in newborn with NE who received TH was associated with death and abnormal neurodevelopmental outcome at 12 months of age [19].

Tumor Necrosis factor (TNF-β) is another pro-inflammatory cytokine which acts on different cells. It promotes the proliferation of fibroblasts and induces the synthesis of GM-CSF, G- CSF, IL-1, and prostaglandin-E2 in fibroblasts [20]. We demonstrated high levels of TNF-β in response to LPS stimulation in school age children with NE. We found an association of high TNF-β levels in school age children with NE with low gross motor scores on developmental assessment. Previous studies have demonstrated an association between neonatal serum cytokine levels of IL-8, Il-12, TNFα and TNF-β in extremely low birth weight infants and Cerebral Palsy [21]. But there are no studies to our knowledge, showing an association of poor neurodevelopmental outcome and abnormally elevated cytokine levels at school age in children with NE.

Interleukin-10 (IL-10) is an anti-inflammatory cytokine, due to its ability to inhibit macrophage activation. It also has a stimulatory function on B cells and mast cells. Increased Il-10 levels were noted in children with NE compared to controls. High levels of IL-10 were associated with severity of NE and in those who received therapeutic hypothermia (TH). IL-10 also has neuroprotective properties demonstrated in animal studies [22].Correlation of high levels of IL-10 with multiorgan dysfunction & mortality has been demonstrated in children in paediatric intensive care [23]. Neonates with sepsis, pneumonia and necrotising enterocolitis demonstrated high levels of Il-10 [24]. Increased levels of IL-10 at 72 h of age were associated with prolonged NICU stay in neonates with Transposition of great arteries who underwent the arterial switch procedure [25].

Erythropoietin (Epo) is a glycoprotein cytokine secreted by the kidney in response to cellular hypoxia. It stimulates the production of red blood cells from the bone marrow. Apart from being an antioxidant, it also has anti-apoptotic and anti-inflammatory actions. Elevated Epo levels were demonstrated in children with NE compared to controls, especially in response to LPS. Significantly high levels of Epo have been demonstrated in infants with increased severity of NE and who underwent TH at day 2–4 of life. NE grade II/III and death were associated with elevated Epo levels on Day 3 of life [2, 18]. High levels of serum Epo may represent markers of severity of encephalopathy and brain injury, this theory can be supported by previous studies reported in preterm infants [26].

TNF-α is a proinflammatory cytokine which plays a critical role in the local inflammatory response and in initiation of the cytokine cascade [27]. TNF-α is an important cytokine involved in sepsis and inflammation and is a potential marker in the diagnosis of early and late onset neonatal sepsis. Increased serum level of TNF-α has been reported to be associated with mortality and abnormal neuroimaging in neonates with NE [18]. In our study we demonstrated significantly increased TNF-α in response to LPS in school age children in the NE and the control groups.

We have shown in our study that school age children with NE demonstrated vigorous systemic innate immune response compared to children with normal development. Periventricular leukomalacia (PVL) or related inflammation events or both during the perinatal and postnatal period may have a programming effect, causing altered inflammatory responses in preterm children with CP [28]. In their study they demonstrated that preterm school-age children with periventricular leukomalacia (PVL) induced (CP) had significantly higher levels of tumour necrosis factor (TNFα) and elevated TLR4 mRNA in peripheral blood mononuclear cells (PBMC’s), in comparison to preterm term school-age control group children.

Lipopolysaccharide (LPS) when introduced in vitro in healthy individuals not only induces the cascade of inflammatory pathways [29] but also initiates a transient refractory state, referred to as LPS hypo-responsiveness or LPS tolerance [30]. This LPS tolerance state is associated with decreased capacity of whole blood and peripheral mononuclear cells to produce proinflammatory cytokines in response to LPS stimulation. Decreased levels of proinflammatory cytokines IL-6 and TNF-α in the serum and in the peripheral mononuclear cells (PBMC) supernatant are found in response to LPS stimulation in critically ill patients in ICU in comparison to healthy individuals [31]. Similar results were seen in our study, the level of TNF-α rose with LPS stimulation to a lesser degree than in children with NE compared to age-matched controls. In addition, LPS hypo responsiveness was found in children with NE in cytokines IL-6 and TNF-β. Similarly, children in the TH group demonstrated decreased response to LPS in cytokines IL-6, IL-8, IL- 1α, Il-1β, TNF-α and VEGF, which correlated with severity of NE.

There are very few studies in the literature demonstrating the above finding in school age children with NE, we do find studies done in the neonatal period but not at school age. However further research studies and large randomised control trials are required to validate these findings.

## Conclusion

Neonatal Encephalopathy remains an important cause of mortality and long-term neurodevelopmental impairment including Cerebral Palsy. Hypoxic ischaemic injury induces an inflammatory response involving excessive cytokine production. We demonstrated significant alteration in cytokines in children with NE at school age compared to normal children which supports our hypothesis of a persistent inflammatory response years after hypoxic ischaemic injury. We also showed an association between elevated proinflammatory cytokine TNF-β and poor neurodevelopmental outcome using ASQ-3 questionnaire. Thus demonstrating that proinflammatory cytokines may act as important biomarkers in prediction of neurodevelopmental outcome in children with NE. Persistent inflammatory response seen in children with NE at school age indicates a longer “therapeutic window” may be available for adjunctive therapies following therapeutic hypothermia.

## Data Availability

The datasets used and/or analysed during the current study are available from the corresponding author on reasonable request.

## Abbreviations

CP: Cerebral palsy
EPO: Erythropoietin
GM-CSF: Granulocyte-macrophage colony-stimulating factor
IFN: Interferon
IL: Interleukin
LPS: Lipopolysaccharide
NE: Neonatal Encephalopathy
TNF: Tumor necrosis factor
VEGF: Vascular endothelial growth factor
